# Implementing flexible bronchoscopy in least developed countries according to international guidelines is feasible and sustainable: example from Phnom-Penh, Cambodia

**DOI:** 10.1186/s12890-016-0354-6

**Published:** 2017-01-10

**Authors:** Martin Veaudor, Sébastien Couraud, Sophors Chan, Chanraksmey Choun, Pisethmorokoth Keo, Virginie Avrillon, Pierre-Jean Souquet, Chanty Ny

**Affiliations:** 1 Service de Pneumologie Aigue Spécialisée et Cancérologie Thoracique, Centre Hospitalier Lyon Sud, Institut de Cancérologie des Hospices Civils de Lyon, 69495 Pierre Bénite, France; 2 Association PRUPET, Centre Hospitalier de Moulin Yzeure, Moulins, France; 3 EMR 3738 Ciblage thérapeutique en oncologie, Faculté de médecine et de maïeutique Lyon Sud Charles Mérieux, Université Lyon 1, Villeurbanne, France; 4 Service de pneumologie, Hôpital Preah Kossamak, Phnom Penh, Cambodia

**Keywords:** Bronchoscopy, Cambodia, Guidelines, Least Developed Countries, Pulmonary medicine

## Abstract

**Background:**

Flexible bronchoscopy is pivotal for the diagnosis of most respiratory diseases. A flexible bronchoscopy unit (FBU) was created in 2008 in the Preah Kossamak university hospital (Phnom Penh, Cambodia) through a cooperation program between a French and a Cambodian team. In 2009 we conducted an assessment of the compliance of the FBU to international standards and found that most of French and British guidelines were fully applied or adapted to local practice.

The aim of the current work was to assess FBU again 6 years later, in order to determine if compliance to international guidelines was sustainable.

**Methods:**

The 2015 evaluation was conducted identically to 2009. All recommendation items from the French and the British Thoracic Societies guidelines were assessed individually. Each recommendation was assigned a status expressing the level at which it was respected in Cambodia: *applied, adapted, not applied* and *not evaluable*.

An endoscope microbial sampling was performed as recommended by the French Ministry of Health.

**Results:**

Between 2009 and 2015, the pattern of international recommendations in the Cambodian FBU did not change. Notably the rates of *applied* French evaluable recommendations remained stable: respectively 58% vs 57%. Main changes in French guidelines occurred in adapted items that became applied (*n* = 5/15) while 4 previously adapted/applied items became not applied. Furthermore, all microbial analyses showed sterile results.

**Conclusions:**

Our results show that implementation of a high quality FBU in a least-developed country is feasible. In addition, the performance is maintained in the long-term.

**Electronic supplementary material:**

The online version of this article (doi:10.1186/s12890-016-0354-6) contains supplementary material, which is available to authorized users.

## Background

Cambodia remains one of the poorest countries in the world: in 2014, it ranked 144^th^ (of 188 countries) on the Human Development Index [1]. Lower respiratory tract (LRT) infections are a major public health problem in Cambodia. In 2013, according to the World Health Organization, it had the highest prevalence of documented tuberculosis cases in the world, i.e., 715 cases per 100,000 inhabitants [2]. Furthermore, LRT infections are the second leading cause of premature death in Cambodia [3].

Flexible bronchoscopy (FB) is pivotal for the diagnosis of most respiratory diseases and especially those of infectious or neoplastic origin [4]. However, the deployment and use of FB necessitate advanced equipment and competencies. Through to the mid-2000s, Cambodia had no flexible bronchoscopy unit (FBU) meeting international standards in its public hospitals.

There was however an inter-hospital cooperation program between the Lyon Sud Hospital (Lyon, France) and the Preah Kossamak Hospital (Phnom Penh, Cambodia) [5]. Building upon that ongoing program, a decision was made in 2008 to create a modern FBU in Cambodia.

Toward this goal, two Cambodian physicians had been brought to France for a 2-year training program, and two residents from the Lyon Sud Hospital spent 6 months in Cambodia (between November 2008 and May 2009) to aid the local team with the deployment of the FBU. The materials (endoscope, light source, camera) were provided as a donation by the Lyon University Hospital team. A Cambodian nurse was hired specifically for the FBU and trained during 2 weeks in France then 6 months in Cambodia.

The FBU was designed to conform as much as possible to current international recommendations while nonetheless taking into consideration local sanitary, economic and organizational constraints and conditions. Following upon the launch of the FBU in 2009, we performed and published an evaluation of the unit’s first-year conformity to international standards [6]. 54 procedures were performed in 2009. The results of that first study showed that it is possible to deploy a high quality FBU in a least-developed country: we showed that 52% of French recommendations [7] and 46% of British recommendations [8] were strictly applied, whereas respectively only 18 and 23% could not be applied.

Now up and running, the remaining major challenges for the FBU are the appropriation of its daily operation by local teams and the perpetuation of quality standards and optimal use. Considering this, a follow-up study to evaluate the long-term respect of recommendations appeared necessary.

Thus, for the present work, we re-evaluated the FBU to assess its continuing application of international recommendations 6 years after its launch.

## Methods

### Evaluation protocol

The protocol used for this second evaluation performed in 2015 (2015 evaluation) was the same as that used for the first evaluation in 2009 (2009 evaluation) [6] to enable a comparison of the results of the two evaluations; only the on-site observer (MV) differed. For the present study, like for the first, the on-site observer was a Lyon Sud Hospital resident assigned for 6 months to the Preah Kossamak Hospital in Phnom Penh as part of the cooperation program between the two hospitals. All examinations performed in the FBU between November and December 2015 (*n* = 14) were observed by the resident to assess the application of recommendations. The medical and paramedical teams were not informed of the observation process until after the observation period to avoid bias.

As in the 2009 evaluation, all currently-applicable recommendations were assessed individually. Each recommendation was assigned a status expressing the level at which it was respected:

- *Applied* when the recommendation was entirely respected;
- *Adapted* when the recommendation was not applied as written but as adapted to respond to local conditions or constraints;
- *Not applied* when the recommendation was not respected at all;
- *Not evaluable* when the recommendation could not be evaluated in the setting of FB in Cambodia.

### Recommendations used for the assessment

Two sets of recommendations were used as practice standards: those published by the Francophone Endoscopy Group of the Francophone Society of Pulmonology (SPLF) [7] and those published by the *British Thoracic Society (BTS)* [8]. Both documents are similarly structured, with lists of recommendations grouped into a number of specific sections.

The BTS recommendations used in the 2009 evaluation were those first published by the society in 2001 [8]. The BTS updated their recommendations in 2013 [9]. The initial and updated BTS recommendations differ on several points. For the new version, the organization of the existing sections was largely modified, new details were added to some subjects (standards and performances of diagnostic techniques, FB in intensive care units, cleaning and disinfection) and new sections were added (sedation). Some items were reworded or modified. For example, the 2001 version recommended antibiotic prophylaxis in certain conditions (notably for patients with heart valve prosthesis) but the 2013 version does not. Recommendations for cleaning and disinfection also evolved, with the 2013 version insisting on the use of automated endoscope reprocessors (AERs) and disposable materials. For the present 2015 evaluation, we logically chose to use the 2013 version of the BTS recommendations.

The second set of recommendations, those published by the SPLF in 2007 [7], had not been updated as of the launch of the present follow-up study.

To ensure the pertinence and clarity of comparisons with, on one hand, the results from the 2009 evaluation and, on the other, the unchanged French recommendations, we chose to reemploy for the 2015 evaluation the presentation of results used in the 2009 evaluation. Thus, the recommendation items are again grouped in six categories: *patient safety* [*before*/*during*/*after*] *endoscopy*; *cleaning and disinfection*; *staff safety*; and *standards and performances of diagnostic techniques*. BTS items that were substantially modified between evaluations were marked with an asterisk in the tables of the present study.

Some items present in the recommendations were evaluated neither in the 2009 evaluation nor in the present 2015 evaluation due to economic and organizational conditions that prevent the development and implementation of certain acts. For example, recommendations concerning interstitial pathologies were and remain unevaluable. Also, the 2013 version of the BTS recommendations has a new section on sedation. However, the safety conditions necessary for the intravenous sedation that it proposes are currently not available in Cambodia, and thus this section was not evaluated here. Finally, in 2009, we had deployed a protocol for FB in the intensive care unit. However, that protocol had fallen out of use several years before the 2015 evaluation and thus was not re-evaluated in it.

### Microbiological analysis of endoscopes

Endoscope microbial surveillance was performed in accordance with the recommendations of the French Ministry of Health [10]. As specific sampling solutions were not available in Cambodia, we used sterile normal saline (0.9% sodium chloride) as suggested in those recommendations. All three of the endoscopes used at the FBU were controlled as per the global channel evaluation method indicated in the above-mentioned recommendations. A sterile syringe was used to inject the normal saline in all the channels of the endoscope and the resulting flush was passively collected in a unique container. Thereafter, the on-site observer took the samples immediately to the Institut Pasteur in Cambodia for microbiological analyses. Mycobacteria were assessed first by microscopy using Ziehl-Neelsen staining then by culturing both in Löwenstein–Jensen medium and in mycobacteria growth indicator tubes. Cyto-bacteriology was done by cell count (epithelial cells/leukocytes) followed by direct bacteriological and mycological examination and finally bacterial culture on standard media.

### Statistical analyses

The number of *applied*, *adapted*, *not applied*, and *not evaluable* items in the French recommendations and in the British recommendations as well as in the 2009 evaluation and in the 2015 evaluation was expressed as a percentage of the total number of recommendations or the number of evaluated recommendations (total - non-evaluable items).

Proportions between the 2009 and 2015 evaluations were compared with the Chi-squared test with n-1° of freedom (*n* = number in the category) when the expected frequency was greater than five or with a two-tailed Fischer exact test when it was not. Statistical significance was set at *p* < 0.05.

## Results

### Application of recommendations

**Fig. 1:**
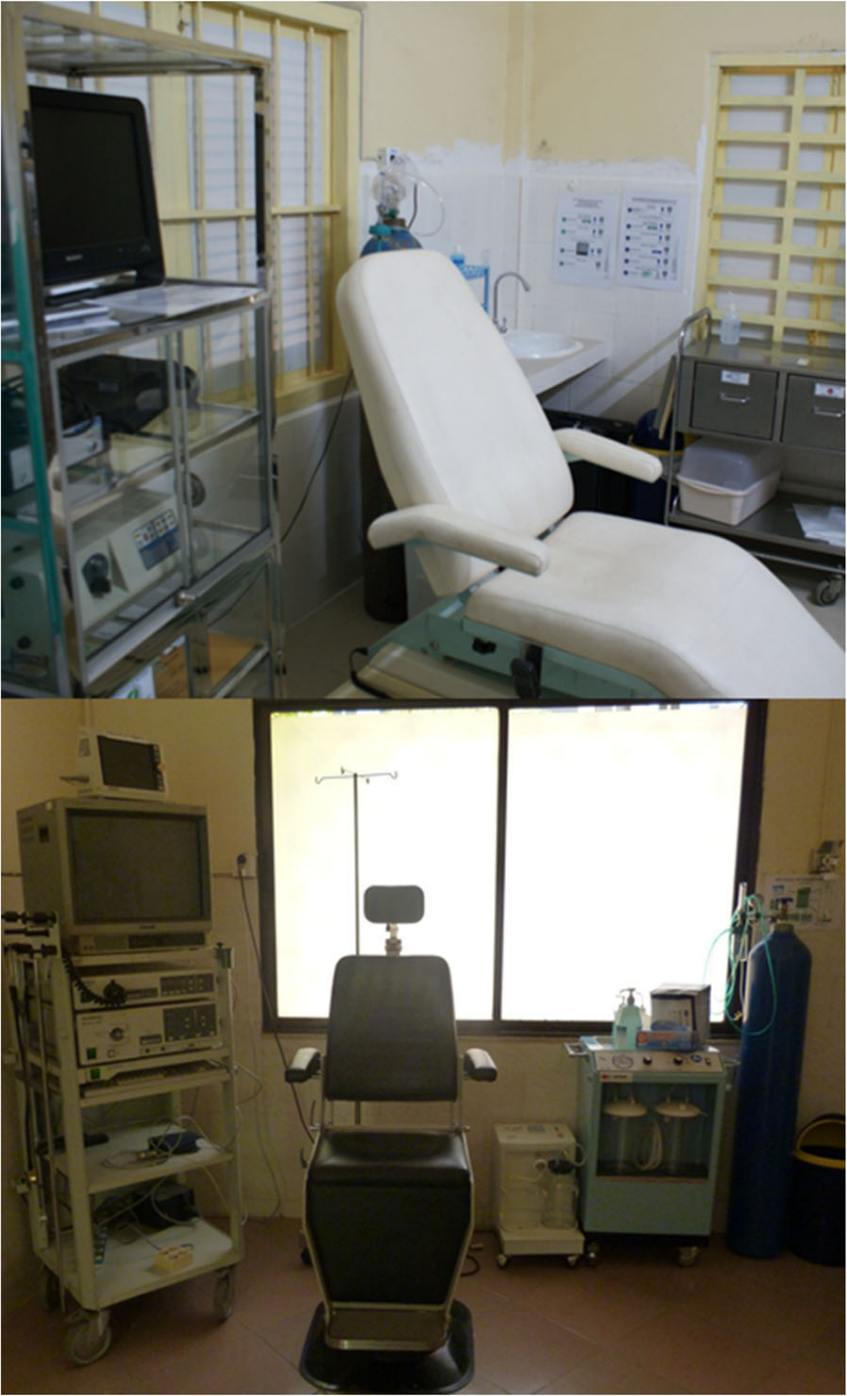
The bronchoscopy unit at the Preah Kossamak Hospital in Phnom Penh in 2009 (*Up*) and 2015 (*Down*)

Figure 1 provides photos of the FBU at the Preah Kossamak Hospital in 2009 and 2015.

Table 1 summarizes the frequencies of recommendation statuses per category and in total for each set of guidelines. Comparing the statuses of the French guidelines between 2009 and 2015, respectively 72% vs 55% were *applied* or *adapted*, 18% vs 20% were *not applied* and 9% vs 25% were *not evaluable*. In 2015, items in the categories *patient safety before* and *patient safety during endoscopy* were *applied* in respectively 63 and 78% of cases. As for the statuses of the British guidelines, 46% of the recommendations were *applied* in 2009 vs 35% in 2015, and 9% were *not evaluable* in 2009 vs 41% in 2015.

**Table 1:** Results of the 2009 and 2015 evaluations for the then-current French and British recommendations

Recommendations	French guidelines			British guidelines		
Year of evaluation	2009 N (%)	2015 N (%)	*P* value	2009^a^ N (%)	2015^b^ N (%)	*P* value
Before bronchoscopy						
Applied	11 (69%)	10 (63%)	-	9 (64%)	18 (78%)	-
Adapted	2 (12%)	2 (12%)		1 (7%)	3 (13%)	
Not applied	2 (12%)	1 (6%)		3 (21%)	0 (0%)	
Not evaluable	1 (6%)	3 (19%)		1 (7%)	2 (9%)	
Total	16	16		14	23	
During bronchoscopy						
Applied	8 (89%)	7 (78%)	-	5 (50%)	10 (59%)	-
Adapted	1 (11%)	1 (11%)		2 (20%)	1 (6%)	
Not applied	0	1 (11%)		2 (20%)	4 (23%)	
Not evaluable	0	0 (0%)		1 (10%)	2 (12%)	
Total	9	9		10	17	
After bronchoscopy						
Applied	1 (25%)	1 (25%)	-	1 (20%)	1 (20%)	-
Adapted	1 (25%)	0		2 (40%)	0	
Not applied	0	0		0	1 (20%)	
Not evaluable	2 (50%)	3 (75%)		2 (40%)	3 (60%)	
Total	4	4		5	5	
Cleaning and disinfection						
Applied	4 (33%)	4 (33%)	-	5 (33%)	8 (31%)	-
Adapted	3 (25%)	1 (8%)		4 (27%)	1 (4%)	
Not applied	5 (42%)	7 (58%)		6 (40%)	11 (42%)	
Not evaluable	0	0		0	6 (23%)	
Total	12	12		15	26	
Staff safety						
Applied	3 (30%)	4 (40%)	-	4 (36%)	3 (27%)	-
Adapted	3 (30%)	2 (20%)		3 (27%)	0	
Not applied	4 (40%)	4 (40%)		4 (36%)	5 (45%)	
Not evaluable	0	0		0	3 (27%)	
Total	10	10		11	11	
Standards and performances of diagnostic techniques						
Applied	1 (14%)	2 (28%)	-	0	5 (28%)	-
Adapted	3 (43%)	2 (28%)		2 (50%)	2 (11%)	
Not applied	0	0		0	4 (22%)	
Not evaluable	3 (43%)	3 (43%)		2 (50%)	7 (39%)	
Total	7	7		4	18	
Intensive care unit						
Applied	6 (86%)	0	-	6 (100%)	0	-
Adapted	0	0		0	0	
Not applied	1 (14%)	0		0	0	
Not evaluable	0	7 (100%)		0	17 (100%)	
Total	7	7		6	17	
Sedation						
Not evaluable	-	-	-	-	13 (100%)	-
Total					13	
Total	*n* = 65	*n* = 65	0.64^#^	*n* = 65	*n* = 130	0.062^#^
Applied	34 (52%)	28 (43%)		30 (46%)	45 (35%)	
Adapted	13 (20%)	8 (12%)		14 (22%)	7 (5%)	
Not applied	12 (18%)	13 (20%)		15 (23%)	25 (19%)	
Not evaluable	6 (9%)	16 (25%)	0.095^$^	6 (9%)	53 (41%)	<10^−4^^$^

% values are calculated within all status (including not-evaluable)

^a^Calculated using 2001 edition of BTS guidelines/^b^Calculated using 2013 edition of BTS guidelines

^#^ *P* value calculated excluding the *not evaluable* status; ^$^ *P* value calculated for all statuses (included *not evaluable* status)

Tables S1 through S6 in the Additional file 1 provide the statuses for all the recommendations, grouped within their categories, for both the 2009 and the 2015 evaluations.

Table 2 summarizes the French recommendations for which there was a status change between the 2009 and 2015 evaluations.

**Table 2:**
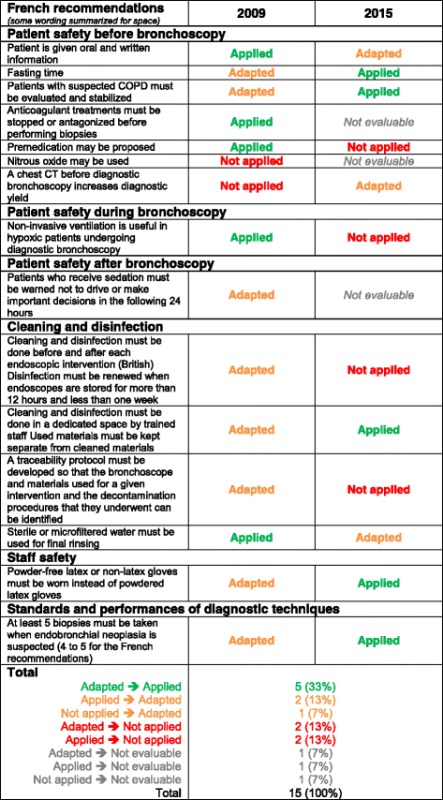
Focus on changes of statuses of the French recommendations in the 2009 and 2015’ evaluations

**Fig. 2:**
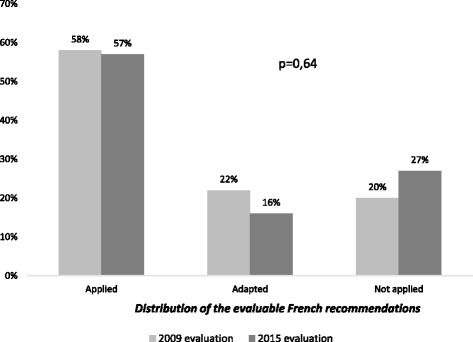
Changes in the distribution of the evaluable French recommendations between 2009 and 2015

Figure 2 summarizes changes in the distribution of the evaluable French recommendations (excluding thus the *not evaluable* status) between the two evaluations: 57% of recommendations were *applied* in 2015 compared to 58% in 2009 (*P* = 0.64).

### Microbiological analysis of endoscopes

All three endoscopes passed the microbial surveillance analyses. Particularly, cytology found less than 10 epithelial cells and less than 10 leukocytes per milliliter. The direct bacteriological and mycological examinations were negative as were the ensuing bacterial cultures on standard media. Similarly, mycobacteriological analyses (microscopy and culture) were negative for all samples.

## Discussion

Between the 2009 and the 2015 evaluations, the profile for the use of international recommendations in the FBU of the Preah Kossamak Hospital did not significantly change. Notably the rates of *applied* French recommendations remained stable: respectively 52% vs 43%. Furthermore, all microbial surveillance analyses were negative for all of the endoscopes.

To our knowledge, ours study is the first to provide data on recommendation application rates, be it in developing or developed countries. Our work here falls within a dynamic of improvement of care provision quality and aligns with the recommendation for periodic audits of FBUs expressed in the 2013 BTS guidelines.

Several changes in the implementation of recommendations between 2009 and 2015 are to be noted. Considering *patient safety during endoscopy*, the recommendation for having non-invasive ventilation available was *applied* in 2009 but not in 2015. Although the equipment is available, the team lacks training for it and thus it remains unused. Also, certain *cleaning and disinfection* recommendations that had the *adapted* status in 2009 were no longer being implemented in 2015. This was the case for renewed disinfection after prolonged storage of an endoscope, abandoned due to costs and a lack of trained staff. However, this had no apparent impact on endoscope hygiene as illustrated by the good microbial surveillance results. Inversely, the recent acquisition of a new multi-detector CT by the hospital enables pre-bronchoscopy thoracic scans, but the examination is prohibitively expensive (US$100) for many patients. The recommendation concerning CT thus evolved from *not applied* to *adapted*.

The lack of an increase in the rate of *applied* recommendations also appears to point to a lack of spontaneous improvement of practices between 2009 and 2015. We underline however that a status of *adapted* or *not applied* for a given recommendation was usually due to elements independent of the pulmonology service such as the ambient poverty or the organization of the healthcare system. The team itself thus has little leeway to further improve the respect of recommendations.

A major issue in cooperation efforts with least-developed countries is the appropriation of projects by the local teams, a step vital to their durability and effectiveness [11]. In the present work, this appropriation appears to have been attained in 2015: the Cambodian team continued to implement international FB recommendations at a rate comparable to that observed at the launch of the project in 2009, and the number of bronchoscopies per month had increased from 4.5 in 2009 [6] to 7 in November-December 2015. This successful appropriation extends to sanitary issues as well, with the continuing and complete respect of recommendations in the *cleaning and disinfection* category, as illustrated by the good results for microbial surveillance. This latter can be improved by making it systematic and more frequent but cost remains an issue: microbial surveillance analyses are billed at US$50/endoscope whereas the bronchoscopy itself is billed at US$20 (excluding analyses) to the patient. This quality control issue appears essential and will be implemented in the future.

Our study has both strengths and weaknesses. Among the former is the long follow-up for the project. Over the 6-year period, the Cambodian FBU experienced numerous organizational changes that had the potential to negatively affect the quality of practices: the unit was moved, a new endoscopic nurse was trained and most of the original endoscopists were replaced by new ones. But these events proved to be opportunities. For example, the change of premises enabled improvements to the FBU, with notably the creation of a dedicated decontamination zone immediately next to, but separated from, the examination room. Equally, the peer-to-peer training of the new nurse by the original nurse, who had been trained at the start of the program, was a chance to verify the local team’s complete comprehension of FB processes and their ability to transmit that knowledge to new staff. The main weakness of our study resides in the changes made to the BTS guidelines between the 2009 and 2015 evaluations. Consequently, numerous items could not be re-evaluated. Interestingly however, a good proportion of the new items in the 2013 BTS recommendations had already been implemented in the FBU upstream of the 2015 evaluation, illustrating the team’s ability to adapt to change. Another weakness is the single-observer nature of the study, which creates a possibility of interpretation and writing biases. And finally, our study is limited by the high number of recommendations that were *not evaluable*. This problem is largely due to the fact that recommendations from developed countries impose increasingly expensive materials (AERs, single-use accessories, etc.) and address indications and techniques that are not currently relevant to Cambodia. For example, such techniques as transbronchial biopsy or fluoroscopy are not yet available in the Preah Kossamak FBU.

## Conclusions

Our results show that it is possible not only to create a high quality flexible bronchoscopy unit in a least-developed country, but also, and particularly, to maintain its performance in the long-term. To succeed in this endeavour, international standards and recommendations must often be adapted to local constraints. More than the cost of modern endoscopic equipment, it is the appropriation of techniques and practices by the local teams that is the largest challenge. Over the 6 years between our first and second evaluations, the Preah Kossamak FBU team succeeded in maintaining an optimal level of care, thus ensuring the safety of their bronchoscopy patients. In the future, we intend to develop several more advanced techniques (such as blind transbronchial needle aspiration of carinal nodes) and organize regular microbial surveillance of equipment. Our successful program, built upon the pertinent application and adaptation of international recommendations, constitutes a model for other medical cooperation projects.

## Additional file

Additional file 1: Table S1. Patient safety before endoscopy. **Table S2.** Patient safety during endoscopy. **Table S3.** Patient safety after endoscopy. **Table S4.** Cleaning and disinfection. **Table S5.** Staff safety. **Table S6.** Standards and performances of diagnostic techniques. (DOCX 82 kb)

## Abbreviations

AER: Automated Endoscope Reprocessor
BTS: British Thoracic Society
FB: Flexible bronchoscopy
FBU: Flexible Bronchoscopy Unit
LRT: Lower respiratory tract
