# Supplementary material for: Implementing flexible bronchoscopy in least developed countries according to international guidelines is feasible and sustainable: example from Phnom-Penh, Cambodia
Source: BMC Pulm Med. 2017 Jan 10;17:10. doi: 10.1186/s12890-016-0354-6 (PMC5223542; doi:10.1186/s12890-016-0354-6)
Supplement: Additional file 1: Table S1. — Patient safety before endoscopy. Table S2. Patient safety during endoscopy. Table S3. Patient safety after endoscopy. Table S4. Cleaning and disinfection. Table S5. Staff safety. Table S6. Standards and performances of diagnostic techniques. (DOCX 82 kb) [file 12890_2016_354_MOESM1_ESM.docx]

**SUPPLEMENTARY INFORMATION**

***Table S 1 – Patient safety before endoscopy***

***Table S 2 – Patient safety during endoscopy***

***Table S 3 – Patient safety after endoscopy***

***Table S 4 – Cleaning and disinfection***

***Table S 5 – Staff safety***

***Table S 6 – Standards and performances of diagnostic techniques***

***Table S1 – Patient safety before endoscopy***

|  | | **2009**^(6)^ | | | **2015** | |
| --- | --- | --- | --- | --- | --- | --- |
| **Recommendations**  *(Wording may be summarized for both publications)* | | **French**^(7)^ | | **British 2001**^(8)^ | **French**^(7)^ | **British 2013**^(9)^ |
| Patient is given oral and written information | | Applied | | Applied | Adapted | Adapted |
| Fasting time | | Adapted | | Not applied | Applied | Applied |
| Informed consent must be obtained | | - | | Applied* | - | Applied |
| **[N]** Baseline oxygen saturation, lung function and comorbidities must be included in pre-FB workup | | - | | - | - | Applied |
| [BTS 2001] Prophylactic antibiotics should be given to patients who are asplenic, have a heart valve prosthesis, or a previous history of endocarditis.  [BTS 2013] Antibiotic prophylaxis is not warranted before bronchoscopy for the prevention of endocarditis,  fever or pneumonia | | - | | Not applied | - | Applied |
| FB should be avoided in the 4 to 6 weeks following a heart attack | | Applied | | Applied | Applied | Applied |
| **[N]** Work in conjunction with the cardiology team if FB is indicated <6 weeks after a heart attack | | - | | - | - | Not evaluable |
| **[N]** Asthma treatment should be optimized before FB | | - | | - | - | Applied |
| Nebulized bronchodilators should be considered before bronchoscopy for asthmatic patients | | Applied | | Applied | Applied | Applied |
| Patients with suspected COPD must be evaluated and stabilized | | Adapted | | Adapted | Applied | Applied |
| **[N]** Age alone should not be considered a contraindication for FB | | - | | - | - | Applied |
| Perform coagulation studies in patients with risk factors | | Applied | | Applied | Applied | Applied |
| Anticoagulant treatments must be stopped or antagonized before performing biopsies | | Applied | | Applied | Not evaluable | Not evaluable |
| Coagulation studies may be done before transbronchial biopsy | | Not evaluable | | Not evaluable | Not evaluable | - |
| In cases of bronchial or transbronchial biopsy, aspirin may be continued; clopidogrel should be stopped 7 days before the intervention | | Applied | | - | Applied | Applied |
| Check for use of antiplatelets | | Applied | | - | Applied | - |
| **[N]** BAL may be performed when platelets are >20,000/µl. Work in conjunction with the hematology team if transfusion is necessary | | - | | - | - | Adapted |
| In the rare situations necessitating continued anticoagulants, INR should be reduced to <2.5 and heparin started | | - | | Applied | - | - |
| **[N]** Consider risks vs benefits of biopsy | | - | | - | - | Applied |
| **[N]** In the presence of hemoptysis with a normal CT, consider FB in cases of persistent bleeding or high lung cancer risk | | - | | - | - | Applied |
| Oxygen supplementation and sedation must be used cautiously especially for patients with COPD | | Applied | | Applied | Applied | Applied |
| [SPLF – BTS 2001] Premedication may be proposed [BTS 2013} Premedication is not routinely indicated | | Applied | | Applied | Not applied | Applied |
| Nitrous oxide may be used | | Not applied | | - | Not evaluable | - |
| Intravenous access must be established before sedation initiation | | - | | Not applied | - | Applied |
| Nebulized lidocaine is not recommended | | Applied | | - | Applied | Applied |
| Atropine is usually not needed | | Applied | | Applied | Applied | Applied |
| A chest CT before diagnostic bronchoscopy increases diagnostic yield of samples | | Not applied | | - | Adapted | Adapted |
| ***Subtotal*** | | ***n=16 (100%)*** | | ***n=14 (100%)*** | ***n=16 (100%)*** | ***n=23 (100%)*** |
| *Applied* | | ***11 (69%)*** | | ***9 (64%)*** | ***10 (63%)*** | ***18 (78%)*** |
| *Adapted* | | ***2 (12%)*** | | ***1 (7%)*** | ***2 (12%)*** | ***3 (13%)*** |
| *Not applied* | | ***2 (12%)*** | | ***3 (21%)*** | ***1 (6%)*** | ***0*** |
| *Not evaluable* | | ***1 (6%)*** | | ***1 (7%)*** | ***3 (19%)*** | ***2 (9%)*** |
|  |  | |  | | | |

* Not included in the subtotal because grouped within a same item in the 2001 recommendations.

** Item is slightly modified in the 2013 edition of the BTS guidelines.

**[N]** Item is new in the 2013 edition of the BTS guidelines.

***Table S2 – Patient safety during endoscopy***

|  | | **2009**^(6)^ | | | **2015** | |
| --- | --- | --- | --- | --- | --- | --- |
| **Recommendations**  *(Wording may be summarized for both publications)* | | **French**^(7)^ | | **British 2001**^(8)^ | **French**^(7)^ | **British 2013**^(9)^ |
| Two assistants, one of whom must be a qualified nurse, must be available | | Adapted | | Adapted | Adapted | Adapted |
| **[N]** Advanced procedures may necessitate supplementary staff | | - | | - | - | Not evaluable |
| Resuscitation equipment must be readily available | | Applied | | Applied | Applied | Applied |
| Non-invasive ventilation is useful in hypoxic patients undergoing diagnostic bronchoscopy | | Applied | | - | Not applied | - |
| Hemostatic/hemodynamic agents (cold saline, epinephrine solutions, terlipressin, etc.) must be available | | Applied | | - | Applied | - |
| **[N]** in the presence of a high risk of arrhythmia the patient's vitals should be optimized | | - | | - | - | Not applied |
| **[N]** Patients must be continuously monitored (heart rate, blood pressure, oxygen saturation) before, during and after FB | | - | | - | - | Applied |
| ECG monitoring is not obligatory but must be available for certain patients | | Applied | | Applied | Applied | Applied |
| Patients must be monitored by pulse oximetry | | Applied | | Applied | Applied | Applied |
| Oxygen supplementation is required when desaturation is significant or prolonged | | Applied | | Applied | Applied | Applied |
| **[N]** Lidocaine should be used for local anesthesia unless contraindicated | | - | | - | - | Applied |
| The total dose of lidocaine must be limited (9mg/kg / SPLF; 8.2mg/kg / BTS 2001) | | Applied | | Adapted | Applied | - |
| The minimal necessary quantity of lidocaine must be used when instilled using the bronchoscope | | Applied | | Applied | Applied | Applied |
| Gel lidocaine is preferable for nasal anesthesia | | - | | Not applied | - | Applied |
| **[N]** Both cricothyroid and spray-as-you-go techniques are effective in delivering lidocaine to the vocal cords and trachea | | - | | - | - | Applied |
| **[N]** 1% lidocaine should be used for spray-as-you-go administration | | - | | - | - | Applied |
| **[N]** The bronchoscopist must remain vigilant for symptoms of lidocaine toxicity | | - | | - | - | Not applied |
| **[N]** The total lidocaine dose must be known | | - | | - | - | Not applied |
| Sedation should be administered in incremental doses to achieve adequate sedation | | - | | Not applied | - | Not applied |
| Fluoroscopic screening should be considered in patients with localized lung lesions | | - | | Not evaluable | - | Not evaluable |
| ***Subtotal*** | | ***n=9 (100%)*** | | ***n=10 (100%)*** | **n=9** | **n=17** |
| *Applied* | | ***8 (89%)*** | | ***5 (50%)*** | ***7 (78%)*** | ***10 (59%)*** |
| *Adapted* | | ***1 (11%)*** | | ***2 (20%)*** | ***1 (11%)*** | ***1 (6%)*** |
| *Not applied* | | ***-*** | | ***2 (20%)*** | ***1 (11%)*** | ***4 (23%)*** |
| *Not evaluable* | | ***-*** | | ***1 (10%)*** | ***0 (0%)*** | ***2 (12%)*** |
|  |  | |  | | | |

***Table S3 – Patient safety after endoscopy***

|  | | **2009**^(6)^ | | | **2015 ^(6)^** | |
| --- | --- | --- | --- | --- | --- | --- |
| **Recommendations**  *(Wording may be summarized for both publications)* | | **French**^(7)^ | | **British 2001**^(8)^ | **French**^(7)^ | **British 2013**^(9)^ |
| Oxygen supplementation may be necessary for some patients | | Applied | | Applied | Applied | -* |
| Chest x-ray may be done after transbronchial biopsy | | Not evaluable | | Not evaluable | Not evaluable | Not evaluable |
| Patients who undergo a transbronchial biopsy should be warned of the risk of pneumothorax | | Not evaluable | | Not evaluable | Not evaluable | Not evaluable |
| **[N]** Patients should be given written information on the risk of post-bronchoscopy fever | | - | | - | - | Not applied |
| Patients who receive sedation must be warned not to drive or make important decisions in the following 24 hours | | Adapted | | Adapted | Not evaluable | Not evaluable |
| **[N]** A qualified nurse is needed to recover the patient after bronchoscopy | | - | | - | - | Applied |
| Patients who were sedated should by accompanied for their return home | | - | | Adapted | - | - |
| ***Subtotal*** | | ***n=4(100%)*** | | ***n=5 (100%)*** | **n=4** | **n=5** |
| *Applied* | | ***1 (25%)*** | | ***1 (20%)*** | ***1(25%)*** | ***1 (20%)*** |
| *Adapted* | | ***1 (25%)*** | | ***2 (40%)*** | ***-*** | ***-*** |
| *Not applied* | | ***-*** | | ***-*** | ***-*** | ***1 (20%)*** |
| *Not evaluable* | | ***2 (50%)*** | | ***2 (40%)*** | ***3 (75%)*** | ***3 (60%)*** |
|  |  | |  | | | |

** Item already recorded in the patient safety during bronchoscopy table and not duplicated in the 2103 edition of BTS guidelines.*

***Table S4 – Cleaning and disinfection***

|  | | **2009**^(6)^ | | | **2015^(6)^** | |
| --- | --- | --- | --- | --- | --- | --- |
| **Recommendations**  *(Wording may be summarized for both publications)* | | **French**^(7)^ | | **British 2001**^(8)^ | **French**^(7)^ | **British 2013**^(9)^ |
| The biocompatibility of decontamination methods must be verified with the material's supplier | | Applied | | Applied | Applied | Applied |
| Cleaning and disinfection must be done before and after each endoscopic intervention (British) Disinfection must be renewed when endoscopes are stored for more than 12 hours and less than one week (French) | | Adapted | | Adapted | Not applied | Not applied |
| Cleaning and disinfection must be done in a dedicated space by trained staff Used materials must be kept separate from cleaned materials | | Adapted | | Adapted | Applied | Applied |
| Meticulous cleaning with a detergent is the most important initial step in the procedure | | Applied | | Applied | Applied | Applied |
| When 2% glutaraldehyde is used for manual and automated disinfection, immersion for 20 minutes is recommended for bronchoscopes at the beginning and end of a session and between patients | | - | | Adapted | - | - |
| Longer, 60-min immersion times are recommended for known or suspected atypical mycobacterial infections, and HIV-positive patients suspected of *mycobacterium avium* intracellular infection | | - | | Not applied | - | - |
| **[N]** Single-use suction valves should replace reusable valves wherever possible | | - | | - | - | Not applied |
| **[N]** Reusable valves should be used only with one bronchoscope and stored alongside the scope for traceability | | - | | - | - | Not applied |
| **[N]** Single-use accessories should be selected over reusable accessories wherever possible | | - | | - | - | Not applied |
| **[N]** When it is necessary to use reusable accessories they must be cleaned according to the manufacturer’s recommendations | | - | | - | - | Applied |
| **[N]** Tracking of patient use of equipment and cleaning processes must be completed after each use | | - | | - | - | Not applied |
| A traceability protocol must be developed so that the bronchoscope and materials used for a given intervention and the decontamination procedures that they underwent can be identified | | Adapted | | Adapted | Not applied | Not applied |
| **[N]** On the grounds of staff safety, manual disinfection is no longer recommended | | - | | - | - | Not applied |
| Bronchoscopes should be processed in AERs | | Not applied | | Not applied | Not applied | Not applied |
| Automated washer disinfectors must have facilities for disinfecting tanks, immersion trays and all fluid pathways | | - | | Not applied | - | - |
| All rinse water pathways must be accessible for regular, preferably sessional, cleaning and disinfection | | - | | Applied | - | - |
| Some mycobacteria are extremely resistant to glutaraldehyde and a chlorine releasing agent or peracetic acid may have to be used via the water filters | | - | | Not applied | - | - |
| Patients with suspected tuberculosis should undergo bronchoscopy at the end of the list | | Not applied | | Not applied | Not applied | - |
| The use of glutaraldehyde should be avoided | | Not applied | | Not applied | Not applied | Not applied |
| **[N]** Disinfectant times should be those recommended by disinfectant manufacturers | | - | | - | - | Applied |
| Double cleaning is obligatory | | Not applied | | - | Not applied | - |
| Quality of rinse water should be assured When in doubt, the external surface of the bronchoscope should be wiped and the lumen flushed with 70% alcohol […] | | - | | Applied | - | - |
| **[N]** The use of 70% alcohol after final rinse is no longer recommended | | - | | - | - | Applied |
| **[N]** Specific storage chambers are recommended for storing clean bronchoscopes | | - | | - | - | Not evaluable |
| **[N]** Bronchoscopes stored in storage chambers should be reprocessed in accordance with the manufacturer’s recommendations | | - | | - | - | Not applied |
| **[N]** When storage chambers are not available, bronchoscopes must be stored in a hanging position, with sufficient space between instruments | | - | | - | - | Applied |
| **[N]** Bronchoscopes must be cleaned and disinfected before and after placing in carrying cases | | - | | - | - | Not evaluable |
| **[N]** Valves must not be attached to bronchoscopes during storage | | - | | - | - | Applied |
| **[N]** AERs should be self-disinfected at the beginning of each day | | - | | - | - | Not evaluable |
| **[N]** AERs must be validated on instillation and following introduction of new disinfectants according to Health Technical Memorandum 01 (HTM-1) | | - | | - | - | Not evaluable |
| **[N]** Regular testing of AERs and final rinse water for mycobacteria must be carried out according to HTM-01 | | - | | - | - | Not evaluable |
| **[N]** Compatibility of bronchoscopes with disinfectant and AER manufacturers’ instructions should be checked | | - | | - | - | Not evaluable |
| Sterile or microfiltered water must be used for final rinsing | | Applied | | Applied | Adapted | Adapted |
| The employed disinfectant must have mycobactericidal activity | | Applied | | - | Applied | - |
| Bronchoscopy should be avoided when Creutzfeldt-Jakob disease is suspected; if it cannot be avoided, the material must be sequestered. Record keeping/identification of high-risk cases is advised | | Not applied | | - | Not applied | Not applied |
| ***Subtotal*** | | ***n=12 (100%)*** | | ***n=15 (100%)*** | **n=12 (100%)** | **n=26 (100%)** |
| *Applied* | | ***4(33%)*** | | ***5 (33%)*** | ***4 (33%)*** | ***8 (31%)*** |
| *Adapted* | | ***3 (25%)*** | | ***4 (27%)*** | ***1 (8%)*** | ***1 (4%)*** |
| *Not applied* | | ***5 (42%)*** | | ***6 (40%)*** | ***7 (58%)*** | ***11 (42%)*** |
| *Not evaluable* | | ***-*** | | ***-*** | ***-*** | ***6 (23%)*** |
|  |  | |  | | | |

***Table S5 – Staff safety***

|  | **2009**^(6)^ | | **2015^(6)^** | |
| --- | --- | --- | --- | --- |
| **Recommendations**  *(Wording may be summarized for both publications)* | **French**^(7)^ | **British 2001**^(8)^ | **French**^(7)^ | **British 2013**^(9)^ |
| All staff must be vaccinated for hepatitis B and tuberculosis | Not applied | Not applied | Not applied | Not applied |
| **[N]** Medical histories of staff should be recorded, including pre-existing asthma, skin and mucosal sensitivities | - | - | - | Not evaluable |
| **[N]** Pre-employment baseline lung function, such as spirometry, should be measured and recorded | - | - | - | Not evaluable |
| **[N]** Annual lung function measurements, such as spirometry, should be performed for all staff directly exposed to disinfectants | - | - | - | Not evaluable |
| A pre-employment health check-up must be done for all people who will work with aldehydes as per CLIN recommendations The occupational physician must screen lung function periodically and regularly and monitor lung symptoms | Not applied | Not applied | Not applied | - |
| If tuberculosis is suspected, additional precautions must be implemented | Not applied | Not applied | Not applied | Not applied |
| Protective clothing and equipment (aprons, gloves, masks, etc.) must be used by the staff during bronchoscopy | Applied | Applied | Applied | - |
| Powder-free latex or non-latex gloves must be worn instead of powdered latex gloves | Adapted | Adapted | Applied | - |
| Injection needles must not be recapped (and must be eliminated in an appropriate medical waste container as per French recommendations). Single-use materials reduce the risk of infection during medical device cleaning | Adapted | Applied | Adapted | - |
| **[N]** Hypodermic needles/other sharp instruments should not be used to remove tissue samples from biopsy forceps | - | - | - | Applied |
| Disposable accessories reduce the risk of infection during cleaning | - | Adapted | - | Not applied |
| **[N]** Reusable spiked forceps are not recommended | - | - | - | Not applied |
| Ideally, bronchoscopes should be disinfected in a dedicated room with automated ventilation preferably in a closed space to avoid contact with disinfectants | Adapted | Adapted | Adapted | - |
| Staff must wear protective equipment when cleaning and disinfecting (nitrile gloves and aprons, visors, breathing masks, others as needed) to protect themselves from projections, aerosols and vapors | Applied | Applied | Applied | Applied |
| Whenever possible autoclavable or single-use accessories should be preferred to avoid unnecessary exposure to disinfectants | Not applied | Not applied | Not applied | - |
| **[N]** Open troughs of disinfectant are not recommended | - | - | - | Not applied |
| The responsible staff must receive specific training | Applied | Applied | Applied | Applied |
| ***Subtotal*** | ***n=10 (100%)*** | ***n=11 (100%)*** | **n=10 (100%)** | **n=11 (100%)** |
| *Applied* | ***3 (30%)*** | ***4 (36%)*** | ***4 (40%)*** | ***3 (27%)*** |
| *Adapted* | ***3 (30%)*** | ***3 (27%)*** | ***2 (20%)*** | ***-*** |
| *Not applied* | ***4 (40%)*** | ***4 (36%)*** | ***4 (40%)*** | ***5 (45%)*** |
| *Not evaluable* | ***-*** | ***-*** | ***-*** | ***3 (27%)*** |

***Table S6 – Standards and performances of diagnostic techniques***

|  | | **2009**^(6)^ | | | **2015^(6)^** | |
| --- | --- | --- | --- | --- | --- | --- |
| **Recommendations**  *(Wording may be summarized for both publications)* | | **French**^(7)^ | | **British 2001**^(8)^ | **French**^(7)^ | **British 2013**^(9)^ |
| **Cancer** | |  | |  |  |  |
| At least 5 biopsies must be taken when endobronchial neoplasia is suspected (4 to 5 for the French recommendations) | | Adapted | | Adapted | Applied | Applied |
| Multiple types of samples (biopsy, brush, lavage) increase the diagnostic yield of bronchoscopy when endobronchial neoplasia is suspected | | Adapted | | Adapted | Adapted | Adapted |
| A minimal diagnostic yield of 80% (French) to 85% (British) can be obtained for endoscopically visible neoplasia | | Not evaluable | | Not evaluable | Not evaluable | Not evaluable |
| *A chest CT before diagnostic bronchoscopy increases diagnostic yield* | | Adapted | | - | Adapted | -* |
| **Infection in immunocompromised patients** | |  | |  |  |  |
| **[N]** In immunocompromised patients with pulmonary infiltrates and in whom tuberculosis is considered unlikely, BAL alone is usually sufficient to achieve a diagnosis. Transbronchial biopsy may be considered | | - | | - | - | Adapted |
| **[N]** BAL or bronchial washings should be sent for microscopy for acid fast bacteria and for mycobacterial culture in patients with pneumonia | | - | | - | - | Applied |
| **[N]** Post-bronchoscopy sputum could be collected in immunocompromised patients suspected to have TB | | - | | - | - | Not applied |
| **[N]** Trans and endobronchial biopsy for invasive aspergillosis may be avoided if BAL galactomannan test is available | | - | | - | - | Not evaluable |
| **[N]** If invasive aspergillosis is suspected, BAL should be sent for microscopy for hyphae and fungal culture | | - | | - | - | Not evaluable |
| **Infection in immunocompetent patients** | |  | |  |  |  |
| **[N]** Bronchoscopy may be considered in non-resolving or slowly resolving pneumonia, especially for current or ex-smokers | | - | | - | - | Applied |
| **[N]** If bronchoscopy is performed for community-acquired pneumonia, BAL specimens should be sent for legionella PCR and atypical pathogens | | - | | - | - | Not evaluable |
| **[N]** Bronchoscopy may be considered if TB is suspected despite negative sputum smear | | - | | - | - | Applied |
| **[N]** Post-bronchoscopy sputum could be collected in patients suspected to have TB | | - | | - | - | Not applied |
| **[N]** In areas with high TB prevalence, samples should be sent for routine TB cultures for all patients | | - | | - | - | Applied |
| **Interstitial diseases** | |  | |  |  |  |
| Several transbronchial samples (biopsies) should be taken from the same lung in cases of diffuse interstitial disease: 4 to 6 is optimal for granulomatosis diagnosis | | Not evaluable | | Not evaluable | Not evaluable | Not evaluable |
| **[N]** In suspected sarcoidosis, endobronchial biopsy should be considered to  increase the diagnostic yield | | - | | - | - | Not evaluable |
| **[N]** transbronchial biopsy is recommended for the diagnosis of stage II–IV sarcoidosis | | - | | - | - | Not evaluable |
| **Other** | |  | |  |  |  |
| Transbronchial biopsy improves diagnostic sensitivity for mediastinal lesions | | Not evaluable | | - | Not evaluable | - |
| Procedural reporting systems should gather all basic information | | Applied | | - | Applied | - |
| **[N]** Bronchoscopists should maintain a record of their personal diagnostic accuracy for FB | | - | | - | - | Not applied |
| **[N]** All bronchoscopy units should perform audits to evaluate diagnostic performance, complications and patient satisfaction | | - | | - | - | Not applied |
| ***Subtotal*** | | ***n=7 (100%)*** | | ***n=4 (100%)*** | **n=7 (100%)** | **n=18 (100%)** |
| *Applied* | | ***1 (14%)*** | | ***-*** | ***2 (28%)*** | ***5 (28%)*** |
| *Adapted* | | ***3 (43%)*** | | ***2 (50%)*** | ***2 (28%)*** | ***2 (11%)*** |
| *Not applied* | | ***-*** | | ***-*** | ***-*** | ***4 (22%)*** |
| *Not evaluable* | | ***3 (43%)*** | | ***2 (50%)*** | ***3 (43%)*** | ***7 (39%)*** |
|  |  | |  | | | |

** Item already recorded in the patient safety before bronchoscopy table and not duplicated in the 2103 edition of BTS guidelines.*
